# Piroxicam Loading onto Mesoporous Silicas by Supercritical CO_2_ Impregnation

**DOI:** 10.3390/molecules26092500

**Published:** 2021-04-25

**Authors:** Marta Gallo, Luca Serpella, Federica Leone, Luigi Manna, Mauro Banchero, Silvia Ronchetti, Barbara Onida

**Affiliations:** Department of Applied Science and Technology, Politecnico di Torino, Corso Duca degli Abruzzi, 24, 10129 Torino, Italy; luca.serpella@polito.it (L.S.); federica.leone@polito.it (F.L.); luigi.manna@polito.it (L.M.); silvia.ronchetti@polito.it (S.R.)

**Keywords:** piroxicam, supercritical CO_2_, mesoporous silica, SBA-15, Grace Syloid^®^ XDP, incorporation, solubility, NSAID

## Abstract

Piroxicam (PRX) is a commonly prescribed nonsteroidal anti-inflammatory drug. Its efficacy, however, is partially limited by its low water solubility. In recent years, different studies have tackled this problem and have suggested delivering PRX through solid dispersions. All these strategies, however, involve the use of potentially harmful solvents for the loading procedure. Since piroxicam is soluble in supercritical CO_2_ (scCO_2_), the present study aims, for the first time, to adsorb PRX onto mesoporous silica using scCO_2_, which is known to be a safer and greener technique compared to the organic solvent-based ones. For comparison, PRX is also loaded by adsorption from solution and incipient wetness impregnation using ethanol as solvent. Two different commercial mesoporous silicas are used (SBA-15 and Grace Syloid^®^ XDP), which differ in porosity order and surface silanol population. Physico-chemical analyses show that the most promising results are obtained through scCO_2_, which yields the amorphization of PRX, whereas some crystallization occurs in the case of adsorption from solution and IWI. The highest loading of PRX by scCO_2_ is obtained in SBA-15 (15 wt.%), where molecule distribution appears homogeneous, with very limited pore blocking.

## 1. Introduction

**Figure 1:**
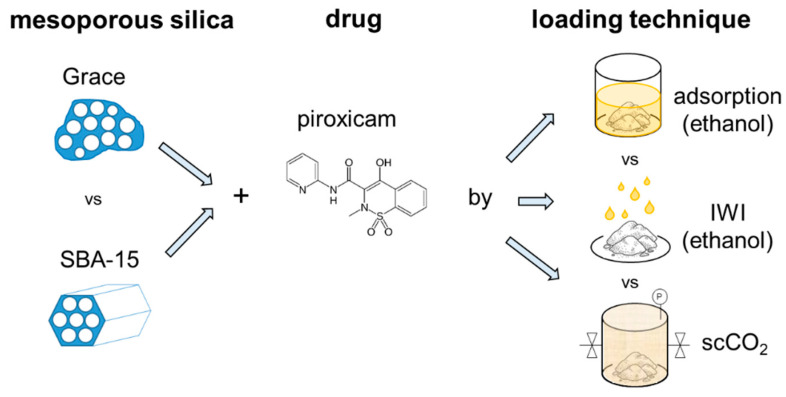
Schematic outline of the experimental study.

Nonsteroidal anti-inflammatory drugs (NSAIDs) are among the most widespread medications used against fever and pain induced by a wide variety of causes (a recent study suggests the use of NSAIDs even as an adjunct therapy against severe COVID infections [1]). Piroxicam (PRX, Figure 1) is an NSAID commonly prescribed to treat both acute and chronic musculoskeletal and joint diseases (e.g., rheumatoid arthritis, osteoarthritis), dysmenorrhea and postoperative pain [2]. Due to its long half-life, piroxicam presents the advantage of requiring one single daily administration [3,4].

Following the Biopharmaceutic Drug Classification System, piroxicam is a class II drug [5], which means that it has high permeability but low water solubility. As a consequence, the absorption of piroxicam is mainly controlled by its dissolution in the stomach and intestine [3]. This is the reason why, in recent years, many studies have been devoted to finding a solution favouring the water solubility of piroxicam. Among the various proposed strategies, many involve the use of solid or semisolid dispersions with different agents, such as polymers, cellulose, surfactant agents, phospholipids [5,6,7,8,9,10,11,12,13,14,15,16]. In all cases, the observed enhancement in PRX solubility may be ascribed to the reduction in the drug particle size, as well as to its being transformed into an amorphous form or to improved wettability, which are both induced by the presence of a hydrophilic carrier. 

Another interesting approach that is also widely used for hydrophobic drugs consists of their dispersion in mesoporous silica supports [17,18]. Due to a confinement effect [19], crystallization is suppressed and the drug in its amorphous form can dissolve more easily when in contact with water solutions. An example with PRX is offered by the study of Ambrogi and coworkers [20]: PRX was loaded from a solvent mixture (acetonitrile/dichloromethane) into MCM-41 mesoporous silica. The high surface area of the system and the amorphousness of PRX increased the drug dissolution rate, especially in acidic conditions where the PRX solubility is lower. Tingming and coworkers [21] also exploited the advantages of mesostructured silica, with the novelty that the surfactant used for the silica synthesis was not removed by calcination. In detail, the SBA-15 precursor still containing the P123 surfactant was loaded with PRX using an acetonitrile/dichloromethane mixture. The results display an interaction between the drug and the surfactant and an improvement in the release kinetics of PRX (burst release), but without a significant increase in solubility.

Although the above-mentioned approaches present interesting advantages, it must be underlined that most of them involve the use of organic solvents such as acetonitrile/dichloromethane [20,21], dichloromethane [16], chloroform [14], and acetone/methanol [9]. When possible, this should be avoided, as organic solvent residuals are undesired in systems that enter the human body. Moreover, the use of organic solvents should be reduced as much as possible in the pharmaceutical industry for the sake of the environment. In this perspective, the use of an alternative impregnation/adsorption solvent such as the supercritical CO_2_ (scCO_2_) is very appealing. In fact, scCO_2_ is green and recyclable since a simple depressurization step allows solvent-free products to be obtained and gaseous CO_2_ to be easily recovered. Indeed, scCO_2_ is diffusely studied in the pharmaceutical field for improving the solubility of class II drugs, taking advantage of techniques such as micronization and polymorphic transformation [22].

According to the literature, the use of scCO_2_ for the incorporation of PRX in solid carriers appears to be still an almost unexplored route and only a few works can be currently found [23,24,25,26,27]. A first example is given by Van Hees and coworkers [23] who studied the solubility of PRX in scCO_2_ and successfully included this drug in β-cyclodextrin, enhancing its water-solubility. A further step was taken by Sauceau [24] and Van Hees himself [25], who added a ternary agent during the incorporation process, which increased the inclusion yield. Additionally, Banchero and coworkers [26] included PRX by means of scCO_2_ in modified cyclodextrin. In all the above-cited cases, scCO_2_ was shown to be a suitable solvent for incorporating piroxicam in cyclodextrins with satisfying inclusion yields (ranging between 66% and 99% depending on the absence or the presence of ternary agents) and improved drug release. ScCO_2_ has also been proposed [27] for impregnating polyvinylpyrrolidone (PVP) with PRX. Interestingly, this work shows that piroxicam, when treated alone with scCO_2_ at temperatures higher than 80 °C, turns into needle crystals, while when it undergoes the same treatment at lower temperatures (e.g., 70 °C) it remains in the same crystalline form (cubic) as the untreated drug. This phenomenon, however, is not observed when the supercritical treatment is conducted in the presence of PVP since this acts as a crystallization inhibitor and the drug turns into an amorphous state, thereby increasing its release kinetics.

Since the above cited works [20,21,23,24,25,26,27] appear to be quite promising for improving PRX bioavailability, the present study aims, for the first time, at merging the advantages of mesoporous silica and scCO_2_ in the preparation of PRX loaded carriers without resorting to organic solvents. For this purpose, two different commercial mesoporous silicas, differing in terms of porous structure and silanols population, were employed (Figure 1): SBA-15, which is a widely studied carrier, and Grace Syloid^®^ XDP, which has already been proved to be a good candidate for drug loading through scCO_2_ [28]. As a comparison, the same supports were also loaded using two traditional techniques: adsorption from solution and incipient wetness impregnation (IWI) (Figure 1). The obtained systems were analysed by thermogravimetric analysis (TGA), nitrogen adsorption isotherms, X-ray diffraction (XRD), Field Emission Scanning Electroscopy (FESEM), and Fourier Transform Infrared (FTIR) spectroscopy. The main objective is to analyze the influence of the loading technique and the support on PRX incorporation in terms of drug content and form (amorphous or crystalline) as well as its interactions with the carrier surface and distribution inside the support. Specifically, this study aims at providing a preliminary evaluation of the PRX incorporation onto mesoporous silica carriers by scCO_2_.

## 2. Results

### 2.1. Thermogravimetric Analyses (TGAs)

**Figure 2:**
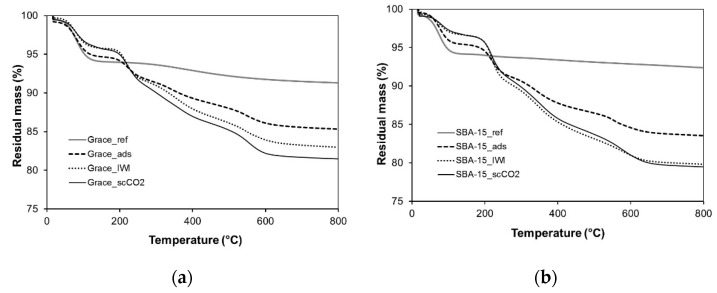
(**a**) Thermogravimetric analysis of Grace reference (Grace_ref) and PRX loaded materials (Grace_ads, Grace_IWI, Grace_ scCO_2_); (**b**) thermogravimetric analysis of and SBA-15 (SBA-15_ref) and PRX loaded materials (SBA-15_ads, SBA-15_IWI, SBA-15_ scCO_2_).

Pristine and impregnated samples underwent TGA. For the pristine supports, this allowed us to evaluate the amount of physisorbed water, which is eliminated below 150 °C, and the mass variation induced by the loss of silanols, which typically takes place above 500 °C [29]. As far as the impregnated samples are concerned, TGA indicated the amount of loaded PRX, which was calculated subtracting the mass loss due to silanols (measured on pristine samples) to the total mass loss between 150 and 800 °C (in order to exclude the contribution of physisorbed water). Results relative to Grace and SBA-15 samples are reported in Figure 2a,b, respectively.

Pristine Grace and SBA-15 silicas (“Grace_ref” and “SBA-15_ref”) present a loss of physisorbed water equal to 6% in mass for both materials and a loss of silanols of 3% and 2%, respectively (Figure 2a,b).

The loss of water in the Grace samples loaded by adsorption from solution (“Grace_ads”), IWI (“Grace_IWI”) and scCO_2_ impregnation (“Grace_scCO_2_”) corresponds to 5%, 4% and 4%, respectively (Figure 2a). For the same samples, at higher temperatures (between 150 and 800 °C) the mass loss is deprived of the silanols’ contribution, which is considered to be constant and equal to that measured for the reference materials (3% and 2% for Grace_ref and SBA-15_ref, respectively). The obtained result is equal to 6.6% for Grace_ads, 10.1% for Grace_IWI and 11.5% for Grace_scCO_2_ and can be ascribed to the amount (% *w*/*w*) of adsorbed PRX.

Analogously, SBA-15 samples loaded by adsorption from solution (“SBA-15_ads”), IWI (“SBA-15_IWI”) and scCO_2_ impregnation (“SBA-15_scCO_2_”) present a mass loss at low temperatures equal to 5%, 3% and 3%, respectively (Figure 2b), whereas between 150 and 800 °C, SBA-15_ads, SBA-15_IWI and SBA-15_scCO_2_ lose 10.1%, 15.0% and 15.3%, respectively, due to the combustion of the adsorbed PRX.

For the sake of clarity, all the above-mentioned results are reported in Table 1.

### 2.2. Nitrogen Adsorption

**Figure 3:**
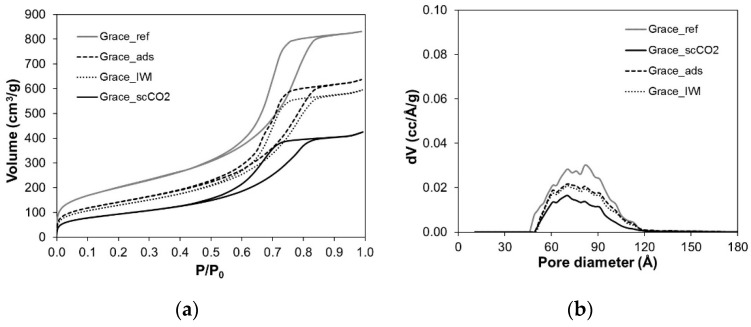
(**a**) Nitrogen adsorption–desorption isotherms of Grace reference and loaded materials; (**b**) corresponding pore size distributions (DFT model).

**Figure 4:**
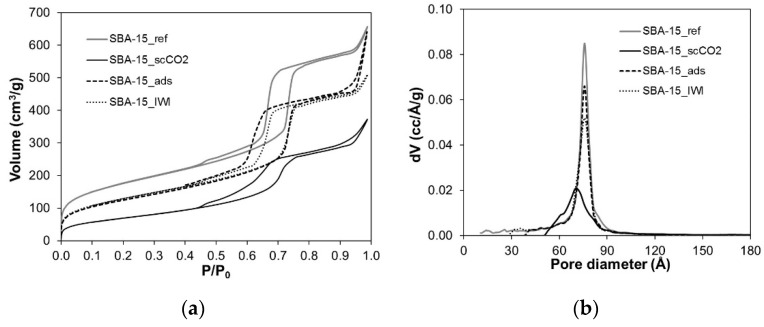
(**a**) Nitrogen adsorption-desorption isotherms of SBA-15 reference and loaded materials; (**b**) corresponding pore size distributions (DFT model).

Nitrogen adsorption isotherms of Grace and SBA-15 silicas, reported in Figure 3a and Figure 4a, provide the value of specific surface area (SSA_BET_), pore volume and pore diameter of the pristine materials as well as the evolution of these parameters due to PRX loading. Figure 3b and Figure 4b present the pore size distribution of the samples and Table 2 reports the parameters obtained by the isotherms.

Both reference materials present an isotherm of type IV according to IUPAC classification (Figure 3a and Figure 4a). The hysteresis loop of Grace_ref can be classified as H2 according to IUPAC classification, which is typical of materials with disordered porosity, whereas the hysteresis loop of SBA-15_ref is the expected H1 type observed for materials with well-defined cylindrical-like pores. Coherently, Grace_ref displays a wider pore size distribution than SBA-15_ref (Figure 3b and Figure 4b), although the average pore diameter is similar (about 76 Å) in the materials. Finally, Grace_ref and SBA-15_ref present SSA_BET_ values and pore volumes of 730 and 640 m^2^/g and 1.29 and 1.02 cm^3^/g, respectively (Table 2).

As far as the loaded Grace samples are concerned, they all still present an isotherm of type IV (Figure 3a), although they show a lower SSA_BET_ value with respect to the unloaded materials (Table 2). Grace_ads and Grace_IWI present similar decreases in the pore volume, while Grace_scCO_2_ displays a significantly higher reduction in this parameter. Accordingly, the decrease in the pore size distribution is similar for Grace_ads and Grace_IWI, whereas it is more evident for Grace_scCO_2_ (Figure 3b). It is worth noting that the pore size distribution decreases in volume but it does not shift towards lower diameter values. Actually, for all the PRX-containing Grace samples, the distribution appears preferentially eroded at values higher than 75 Å.

As far as the loaded SBA-15 samples are concerned, a decrease in SSA_BET_ and pore volume is observed to a smaller extent for SBA-15_ads and SBA-15_IWI and to a greater extent for SBA-15_scCO_2_ (Table 2). Moreover, the pore size distribution changes for SBA-15_scCO_2_, shifting to lower values (Figure 4b), indicating a smaller pore diameter equal to about 70 Å. SBA-15_ads and SBA-15_IWI, instead, do not present any relevant change in the pore diameter (Figure 4b), but only a small decrease in the pore size distribution volume (Figure 4b), in agreement with the pore volume reduction (Table 2).

### 2.3. Field Emission Scanning Microscope (FESEM)

**Figure 5:**
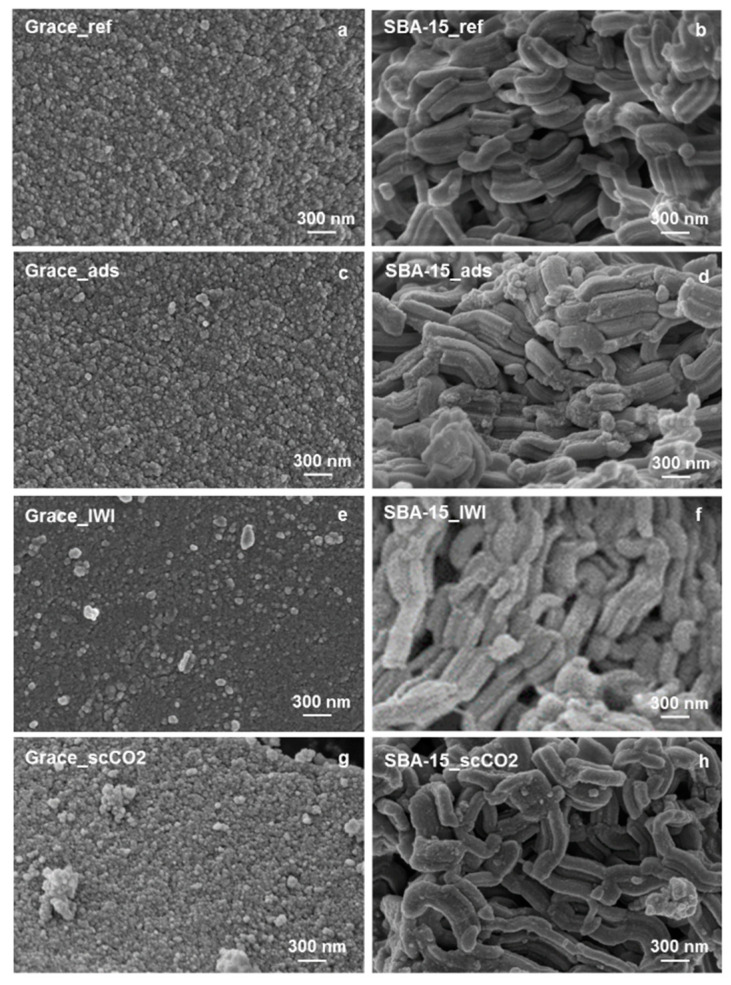
FESEM images of (**a**) Grace_ref; (**b**) SBA-15_ref; (**c**) Grace_ads; (**d**) SBA-15_ads; (**e**) Grace_IWI; (**f**) SBA-15_IWI; (**g**) Grace_scCO_2_; (**h**) SBA-15_scCO_2_.

In Figure 5, the FESEM images of the reference and PRX loaded samples are reported.

Grace_ref appears as an agglomerate of nanoparticles and its mesoporosity may be thus ascribed to interparticle volume (Figure 5a). SBA-15_ref is characterized by the typical elongated particles, which appear to be micron-sized in length (Figure 5b). Samples containing PRX show the same morphology as pristine silicas (Figure 5c–h). 

### 2.4. X-ray Diffraction (XRD)

**Figure 6:**
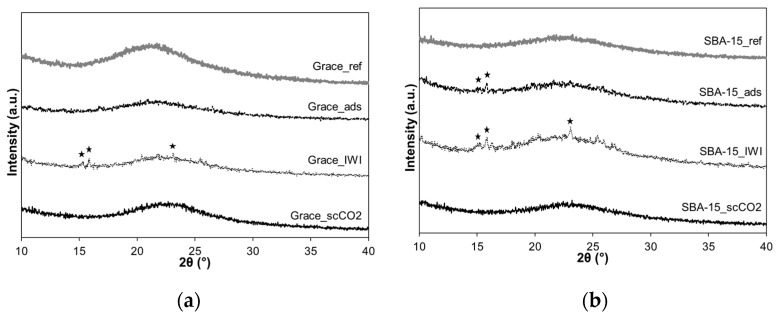
(**a**) XRD patterns of Grace reference and loaded materials; (**b**) XRD patterns of SBA-15 reference and loaded materials (stars indicate peaks of PRX polymorphic form II).

Figure 6 shows the XRD patterns of Grace and SBA-15 reference and loaded materials.

The patterns of the samples loaded by scCO_2_ impregnation (Grace_ scCO_2_ and SBA-15_scCO_2_) exhibit the same diffraction pattern as the Grace_ref and SBA-15_ref and no peaks due to the crystalline PRX are observed. 

At variance with samples loaded by sCO_2_ impregnation, the samples loaded by IWI (Grace_IWI and SBA-15_IWI) show some weak peaks at 2θ equal to 15.3, 15.9 and 23.2 (indicated by stars in Figure 6) related to polymorphic form II of PRX [30]. Weaker peaks of the same polymorphic form are observed for SBA_ads, while they are negligible for Grace_ads. The occurrence of the polymorphic form II is not surprising. Indeed, the polymorphic form II of PRX is obtained by crystallization from solution in absolute ethanol at room temperature [30].

### 2.5. Fourier Transform Infrared (FTIR)

**Figure 7:**
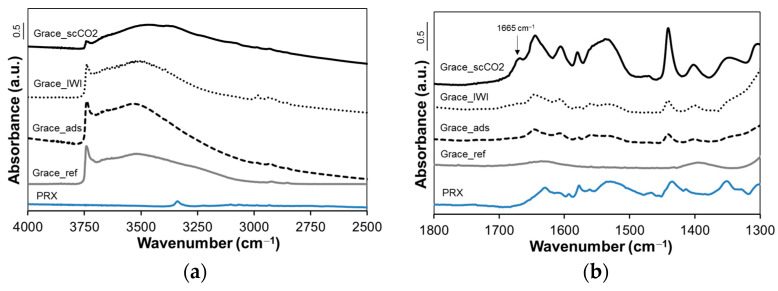
FTIR spectra of crystalline piroxicam, Grace reference and loaded materials in the wavenumber range of (**a**) 4000–2500 cm^−1^; (**b**) 1800–1300 cm^−1^.

**Figure 8:**
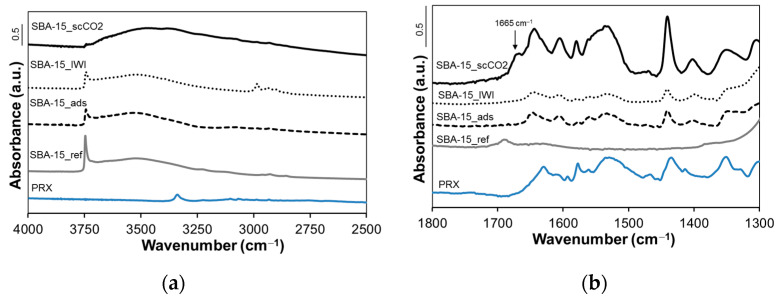
FTIR spectra of crystalline piroxicam, SBA-15 reference and loaded materials in the wavenumber range of (**a**) 4000–2500 cm^−1^; (**b**) 1800–1300 cm^−1^.

Figure 7 and Figure 8 display FTIR spectra of Grace and SBA-15 samples, respectively, together with the spectrum of pure PRX (for comparison). For the sake of clarity, all spectra are reported in two ranges of wavelength: the former between 4000 and 2500 cm^−1^ (Figure 7a and Figure 8a) and the latter between 1800 and 1300 cm^−1^ (Figure 7b and Figure 8b).

For both Grace_ref and SBA-15_ref a peak at 3745 cm^−1^, due to isolated silanols, and a broad band around 3500 cm^−1^, due to interacting silanols, are observed (Figure 7a and Figure 8a). The relative intensity of the band due to isolated silanols is higher for SBA-15_ref, indicating a larger relative population of these surface hydroxyl species in SBA-15_ref than in Grace_ref.

As far as Grace_scCO_2_ and SBA-15_scCO_2_ are concerned, the intensity of the peak due to isolated silanols (3745 cm^−1^) dramatically decreases and a broad absorption appears at lower wavenumbers. This is most evident in the spectrum of SBA-15_scCO_2_, where the intensity of the band due to isolated silanols is negligible.

In the range 1700–1300 cm^−1^ of the spectra of all loaded samples, bands due to PRX molecules are observed. For both silicas, these bands are more intense for samples loaded by scCO_2_ impregnation where, in particular, the appearance of a band at 1665 cm^−1^ is noteworthy (see arrows in Figure 7b and Figure 8b).

## 3. Discussion

In order to fully understand the results obtained with the loaded samples, it is necessary to first analyze the differences and analogies between the two reference materials. 

The TGA results on pristine samples display no relevant difference in the amount of physisorbed water (Figure 2): both Grace_ref and SBA-15_ref lose around 6 wt.% of mass below 150 °C, suggesting a similar hydrophilicity [29]. On the other hand, at higher temperatures (between 150 and 800 °C) a higher mass loss is observed for Grace_ref (3 wt.%) with respect to SBA-15_ref (2 wt.%). This difference can be ascribed to a different content of silanols, which condense above 500 °C [29] so releasing water and causing a mass loss. Indeed Grace_ref is characterized by a higher SSA_BET_ (Table 2) and a higher relative amount of interacting silanols (which are expected to produce condensation upon thermal treatment), as evidenced by the FTIR analyses (Figure 7a), when compared to SBA-15_ref (Figure 8a). 

TGA curves of the loaded materials are comparable to those observed for other mesoporous silica carriers (silica MCM-41 and SBA-15 loaded with PRX) available in the literature [20,21]. After an initial mass loss due to adsorbed humidity (below 150 °C), a gradual mass reduction ascribed to PRX degradation was observed between 150 and 500 °C, which was followed by a final sharper loss (500–550 °C) induced by the final PRX decomposition [20]. With respect to the unloaded materials, when PRX is present, the mass loss is lower at low temperature (below 150 °C), as in the loaded materials the silica surface is occupied by PRX (which is hydrophobic) rather than by molecular water. The higher the PRX content, the more evident this phenomenon is. In particular, the lowest water loss is observed for SBA-15_IWI and SBA-15_scCO_2_, coherent with the fact that in these samples the content of PRX is higher (15.0 wt.% and 15.3 wt.%, respectively) than in the others (all below 12%). In addition, the TGA curves of the loaded materials reach a plateau at different temperatures: around 650 °C for SBA-15_IWI and SBA-15_scCO_2_ and around 600 °C for all the other samples. This could also be due to the dissimilar amount of PRX (Table 1). It is worth noting that the PRX content is coherent with that obtained through scCO_2_ on other supports (e.g., cyclodextrin [26,27]).

Although the Grace silica carrier is characterized by higher SSA_BET_ and pore volume (Table 2), the PRX content is higher in SBA-15 loaded samples than in the corresponding Grace loaded ones, suggesting that the amount of PRX is not surface dependent. It has to be underlined that a possible effect of the different surface polarity of the two silicas on the SSA_BET_ measured by nitrogen adsorption [31] can be considered negligible. A reason for the higher PRX loading obtained with SBA-15 may be found in the uniform porosity of this material (Figure 4b), which favours a homogeneous PRX distribution inside the mesopores. Instead, Grace_ref is characterized by a disordered porosity and a wide pore size distribution, which may be less favourable to diffusion and adsorption of PRX onto the mesopores silica surface. Indeed, after PRX loading the pore size distribution of all Grace samples mainly decreases in volume and changes shape (Figure 3b), even though it does not shift to lower pore size. On the other hand, the pore distribution of SBA-15_scCO_2_, in addition to decreasing in volume, also shifts to lower diameter values (Figure 4b). This suggests a more uniform distribution of PRX molecules on the surface of mesopores in SBA-15_scCO_2_ compared to all other samples.

Considering the higher PRX content in the samples loaded with scCO_2_, a further consideration can be carried out to compare Grace_scCO_2_ and SBA-15_scCO_2_ samples. In particular, the theoretical volume occupied by PRX per silica gram (V_theor_) is calculated starting from the PRX content measured by TGA and the PRX density available from the literature [32]. This volume can be compared with the volume variation experimentally measured by nitrogen adsorption (V_N2_). The difference between the two volumes (ΔV_excluded_) can be attributed to pore blocking phenomena and, to some extent, to errors in the PRX density value (which is considered equal to the density of the crystalline drug here [32]). The above-mentioned values are reported in Table 3. The discrepancy (ΔV_excluded_) between theoretical (V_theor_) and measured volume decrease (V_N2_) is significantly higher for Grace_scCO_2_ (0.35 g/cm^3^ per silica gram) than for SBA-15_scCO_2_ (0.07 g/cm^3^ per silica gram), suggesting a larger pore-blocking in Grace_scCO_2_. In this case, in fact, since pores are blocked, it can be assumed that the pore volume inaccessible to nitrogen is higher than that actually occupied by PRX.

Eventually, regarding the higher content of PRX in all SBA-15 loaded samples than in the corresponding Grace loaded ones (Table 1), it is worth mentioning that also a role of the different silanols population in the two carriers—i.e., the higher relative amount of isolated silanols observed in SBA-15_ref rather than in Grace_ref— cannot be ruled out. For instance, it is known that vicinal and interacting silanols affect the silica surface hydrophilicity [33,34], whereas isolated silanols have been proposed to be more active in the adsorption of certain species, such as amines [35].

As far as the materials morphology is concerned, it is worth noting that the loading treatment did not induce any change in the supports (Figure 5).

The XRD patterns (Figure 6) point out that PRX was successfully incorporated in amorphous form by means of scCO_2_, whereas evidence of crystallization (polymorphic form II of PRX) has been observed for SBA-15_IWI, SBA-15_ads and Grace_IWI. Concerning Grace_ads, this sample has the lowest content of PRX (6.6 wt.%), which may explain the lack of crystallization.

Interestingly, samples loaded by scCO_2_ impregnation are characterized by the highest drug content (11.5%–15.3%) and PRX is completely amorphous. As mentioned in the introduction section, the amorphous form of the active principle is a key factor for increasing the water solubility of hydrophobic drugs, such as piroxicam, and represents, therefore, a relevant result. Adsorption by scCO_2_ allows a PRX distribution inside the mesopores of silica supports to be obtained, as revealed by the variation of SSA_BET_ and pore volume values (Table 2). Whilst constrained in the mesoporosity, PRX undergoes a “confinement” phenomenon, which stabilizes its amorphous form [19]. This effect takes place in both supports, as revealed by the significant pore volume decrease (Table 2).

The presence of PRX on the surface of silica mesopores in SBA-15_scCO_2_ and Grace_ scCO_2_ is revealed by the FTIR spectra (Figure 7 and Figure 8). In particular, the intensity of the peak due to isolated silanols (3745 cm^−1^) significantly decreases after drug loading, whereas absorption ascribed to H-bonded species (below 3500 cm^−1^) increases. This evidence suggests an interaction between PRX and the Si-OH groups of the silica surface through hydrogen bonds. The higher the PRX content, the more visible this phenomenon is: the depletion of isolated silanols, in fact, appears to be greatest for SBA-15_scCO_2_ (the sample with the highest PRX content), for which the peak of isolated silanols almost disappears after drug loading. Moreover, this is in agreement with the more homogeneous distribution of PRX molecules inside mesopores, as revealed by the shift of the pore size distribution to a lower pore diameter observed for SBA-15_ scCO_2_ (Figure 4b). A further comment needs to be made about the band at 1665 cm^−1^, which is observed in the spectra of both SBA-15_scCO_2_ and Grace_scCO_2_ and is absent the spectrum of crystalline PRX, where all absorptions appear at lower wavenumbers (Figure 7b and Figure 8b). This band is tentatively ascribed to the stretching mode of the carbonyl groups of PRX molecules on the silica surface and inside mesopores. In fact, in the crystalline forms of PRX the carbonyl groups are involved in intra- and intermolecular H-bonding [36], which are expected to downshift the stretching mode compared to free groups [37]. Therefore, it is proposed here that the lack of the H-bonding network typical of the crystalline form is responsible for the appearance of the stretching mode of carbonyls at higher wavenumbers, which are known for free or *quasi*-free groups [37].

It is worth noting that for SBA-15_ads and SBA-15_IWI, where PRX crystallization occurred, no changes in the pore size were observed when compared to SBA-15_ref (Figure 4b). This is in agreement with the location, at least to some extent, of PRX molecules at the external surface and at the entrance of mesopores, causing pore blocking.

All in all, among the three tested loading techniques, scCO_2_ impregnation is the most promising one in terms of drug content, amorphous form of PRX, homogeneous distribution of the drug and interaction with the carrier surface. Considering the two different supports, SBA-15 can be considered preferable: although this carrier has lower specific surface area and pore volume than Grace, it allows more PRX to be loaded in a more homogenous fashion, probably due to the uniform porosity.

For completeness, results obtained with SBA-15_scCO_2_, which is the most promising among the samples listed here, can be analyzed considering some similar supports presented in the literature. For example, a comparison can be made with the studies by Ambrogi and coworkers [20] and Tingming et al. [21], who loaded PRX on MCM-41 [20] and SBA-15 [21] by adsorption from solution using acetonitrile/dichloromethane as solvent. The drug contents (14% in MCM-41 and 18% in SBA-15) are comparable to that of SBA-16_scCO_2_ (15%). As a conclusion, the comparison with the literature, whilst very limited, shows that the use of scCO_2_ allows effective PRX loading to be obtained avoiding the use of undesired organic solvents.

Finally, as an outlook, considering the relatively limited amount of drug incorporated, the topical field could be a valid use of these carriers. Drug delivery through the skin, in fact, usually implies lower doses compared to oral administration, but a more sustained release during time. In this perspective, the carriers presented in this study (especially SBA-15) could fulfill the requirements of topical piroxicam administration. Therefore, a future release study in conditions mimicking the skin surface could provide interesting information to compare carriers loaded through different techniques and evaluate the effect of PRX crystallization and distribution on the release kinetics.

## 4. Materials and Methods

### 4.1. Materials

For the present study, two commercially available mesosporous silica supports were used: SBA-15 (nominal pore diameter 8 nm, Sigma Aldrich, Milano, Italy) and Grace Syloid^®^ XDP SP53D-11804 (Grace, Columbia, MD, USA). The first material (named “SBA-15_ref” in the manuscript) has an ordered 2D hexagonal symmetry. The second one (named “Grace_ref” for simplicity) presents an interparticle disordered porosity.

### 4.2. Loading Techniques

The loading of piroxicam was performed through adsorption from solution using ethanol as solvent (samples names “Grace_ads” and “SBA-15_ads”), incipient wetness impregnation (samples “Grace_IWI” and “SBA-15_IWI”) and supercritical CO_2_ impregnation (samples “Grace_scCO_2_” and “SBA-15_scCO_2_”). In detail, for the adsorption from solution, one gram of silica was placed in a solution made of 160 mg of PRX in 50 mL of ethanol. After 48 h under stirring at room temperature, the solution was filtered and dried at 40 °C to retrieve the loaded carrier. For the IWI procedure, another solution of PRX in ethanol was prepared and gradually administered to the silica. For each impregnation step, 1.8 mg of PRX dissolved in 1.2 mL of ethanol was added to one gram of the carrier; once the solvent evaporated, the same procedure was repeated. At the end of the loading process, 156 mg of PRX was theoretically added to the silica. Incorporation through scCO_2_ was performed according to the procedure described elsewhere [38]. Briefly, a pellet of carrier (100 mg) and a pellet of piroxicam (100 mg) were placed in a glass cylinder and separated by a filter paper disc. The glass cylinder was introduced in a high-pressure vessel [27,38] where the impregnation took place in a static scCO_2_ atmosphere at constant temperature (120 °C) and pressure (300 bar) for a fixed time (12 h). Temperature and pressure were selected according to a previous work [26] and taking into account that, when it is mixed with a carrier and contacted with scCO_2_, piroxicam may degrade at lower temperatures (120–150 °C) than its melting point (198–200 °C) [26,27]. The contact time was selected in analogy to a previous work related to the supercritical incorporation of clotrimazole in an ordered mesoporous silica. Tests at different contact times showed that a 12 h time could guarantee equilibrium incorporation of the drug [39].

### 4.3. X-ray Diffraction

Physico-chemical characterization of pristine and impregnated carriers was carried out. In detail, XRD patterns were acquired with a Panalytical X’Pert PRO (Cu Kα radiation, Malvern Panalytical, Almelo, The Netherlands). Data collection was performed at 40 kV and 40 mA with a solid-state detector (PIXcel1D) at high angles (2θ = 10–40°).

### 4.4. Nitrogen Adsorption

Nitrogen adsorption isotherms were obtained with a Quantachrome AUTOSORB-1 instrument (Quantachrome Instruments, Boynton Beach, FL, USA). Samples were outgassed at 70 °C for 2 h before the measurements. Specific surface area and pore size distribution were calculated according to Brunauer–Emmet–Teller (BET) and Density Functional Theory (DFT) models, respectively.

### 4.5. Fourier Transform Infrared Spectroscopy

FTIR spectra (resolution of 2 cm^−1^) were recorded with an Equinox 55 spectrometer (Bruker, Billerica, MA, USA) on self-supporting pellets (with the addition of KBr for PRX). Before the measurement, samples were outgassed at room temperature (residual pressure of 0.1 Pa).

### 4.6. Field Emission Scanning Microscopy

FESEM was performed with a Supra 25 instrument (Carl Zeiss, Oberkochen, Germany).

### 4.7. Thermogravimetric Analysis

TGAs were conducted on a Setaram DSC/TGA 92-16.18 (Caluire, France), heating the samples between 20 and 800 °C at a heating rate of 10 °C/min.

## 5. Conclusions

In this work, the loading of piroxicam (PRX) in two mesoporous silicas, i.e., SBA-15 and Grace Syloid^®^ XDP, by means of supercritical CO_2_ impregnation is reported for the first time. For both silicas, amorphization of PRX was achieved and molecules were located inside mesopores, interacting with the internal silica surface through hydrogen bonding. The highest loading was reached using SBA-15 (15 wt.%), despite the lower SSA_BET_ and pore volume of the silica. This is mainly ascribed to its uniform porosity, allowing a homogeneous distribution of PRX inside mesopores to be obtained, whereas some pore blocking is suggested to occur in the Grace Syloid^®^ XDP, characterized by disordered porosity and wide pore size distribution.

Adsorption from solution and IWI, using ethanol as solvent, was studied for comparison, revealing the occurrence of a certain degree of PRX crystallization in the polymorphic form II, despite the lower amount of loaded PRX.

As a whole, the synergy between the scCO_2_ impregnation process and mesoporous silica carriers appears promising for the preparation of PRX delivery systems avoiding the use of undesired organic solvents.

## Figures and Tables

**Table 1:** Mass losses derived from TGA.

Sample	Molecular Water (%)	PRX (%) *
Grace_ref	6.0	-
SBA-15_ref	6.0	-
Grace_ads	5.0	6.6
SBA-15_ads	5.0	10.1
Grace_IWI	4.0	10.1
SBA-15_IWI	3.0	15.0
Grace_scCO_2_	4.0	11.5
SBA-15_scCO_2_	3.0	15.3

* corresponds to PRX content (% *w*/*w*).

**Table 2:** Textural features derived from nitrogen adsorption–desorption analysis.

Sample	SSA_BET_ (m^2^/g)	Pore Volume (cm^3^/g)
Grace_ref	730	1.29
SBA-15_ref	640	1.02
Grace_ads	515	0.99
SBA-15_ads	455	0.99
Grace_IWI	475	0.92
SBA-15_IWI	470	0.79
Grace_scCO_2_	340	0.66
SBA-15_scCO_2_	250	0.58

**Table 3:** Theoretical and measured volume decrease in scCO_2_ loaded samples (per silica gram) and their difference.

Sample	V_theor_ (cm^3^/g) *	V_N2_ (cm^3^/g)	ΔV_excluded_ (cm^3^/g)
Grace_scCO_2_	0.19	0.54	0.35
SBA-15_scCO_2_	0.27	0.34	0.07

* assuming PRX density = 1.481 g/cm^3^.

## Data Availability

The data presented in this study are available in this article.

## Sample Availability

Samples of the compounds are available from the authors.
